# Waugh's Syndrome: A Case Report and Literature Review of Intussusception and Malrotation

**DOI:** 10.1055/a-2781-7827

**Published:** 2026-01-27

**Authors:** Mário Rui Correia, Ana Isabel Barros, Jorge Cagigal, Joana Sinde, Catarina Sousa-Lopes, Maria Luísa Gaspar, Helena M. Silva, Hélder Morgado, José Banquart-Leitão

**Affiliations:** 1 Serviço de Cirurgia Pediátrica, Centro Materno Infantil do Norte Albino Aroso, Centro Hospitalar Universitário de Santo António, Unidade Local de Saúde de Santo António, Porto, Portugal; 2 Unidade de Gastroenterologia Pediátrica, Serviço de Pediatria, Centro Materno Infantil do Norte Albino Aroso, Centro Hospitalar Universitário de Santo António, Unidade Local de Saúde de Santo António, Porto, Portugal

**Keywords:** intussusception, malrotation, Waugh's syndrome, Ladd procedure, ultrasound

## Abstract

Waugh's syndrome, the rare coexistence of intussusception and intestinal malrotation, has rarely been reported in literature, with fewer than 100 cases described globally. Its diagnosis is challenging due to non-specific symptoms of both conditions and the frequent success of non-operative reduction of intussusception, which often results in underdiagnosis of malrotation. We present a case of a 6-month-old boy who developed recurrent vomiting, feeding intolerance, and failure to thrive. Ultrasound imaging revealed ileocolic intussusception and a mobile cecum. Laparoscopy confirmed malrotation, and the patient underwent manual reduction of intussusception, followed by Ladd procedure. The child had an uneventful postoperative recovery without complications. Early diagnosis of Waugh's syndrome requires a high index of suspicion, particularly in recurrent obstructive symptoms where malrotation may be present. Enhanced imaging techniques can facilitate prompt diagnosis and guide appropriate surgical intervention, preventing complications. Clinicians should maintain a high degree of suspicion for Waugh's syndrome to ensure timely and effective intervention.

## Introduction

Waugh's syndrome, which describes the rare coexistence of intussusception and intestinal malrotation, has been reported in fewer than 100 cases worldwide. Named by Breckon and Hadley 1 after Waugh and Lond's 2 first description in 1911, the syndrome is likely underrecognized in clinical practice despite the higher individual prevalence of both intussusception and malrotation. Over the years, literature reviews have compiled these rare cases, but the true incidence and pathophysiology remain unclear. 3 4 The concurrent occurrence of these conditions is often missed, particularly as many cases of intussusception are treated non-operatively without further exploration for underlying malrotation.

In this report, we present a case of Waugh's syndrome in a 6-month-old boy who initially presented with persistent vomiting and feeding intolerance, later complicated by intussusception. This case highlights the importance of a high index of suspicion for malrotation in atypical cases of intussusception. Furthermore, we conducted a comprehensive literature review and compiled all reported cases of Waugh's syndrome, contributing to the growing body of literature on this rare condition.

This case has been reported in-line with SCARE (Surgical CAse REport) criteria 5 adapted to this journal's guidelines. 6

## Case Description

A male infant was born at term via cesarean section after an uncomplicated pregnancy. He had a normal neonatal course and normal neurodevelopment. He was breastfed combined with bottle feeding until the age of 4 months, and weaning was uneventful.

At 6 months of age, he presented with intermittent vomiting following an episode of acute otitis media and bronchiolitis. Multiple emergency visits occurred over a 1-month period, including a 1-week hospitalization due to ongoing postprandial vomiting, occasional diarrhea, and feeding intolerance. Initial assessments attributed the symptoms to viral infections and acute gastroenteritis. Table 1 compiles a timeline of clinical presentation and diagnostic progression. Diagnostic investigation concerning food protein-induced enterocolitis, celiac enteropathy, and metabolic disorders was unremarkable. The head computerized tomography was normal. However, non-bilious vomiting became persistent and was associated with progressive weight loss (∼9%). At this point, concern was raised for mechanical obstruction. The patient underwent abdominal Doppler ultrasound, which revealed an ileocolic intussusception. Ultrasound-guided hydrostatic reduction failed, but the experienced pediatric radiologist identified abnormal colonic positioning and mobility, raising suspicion of malrotation—a finding that might also have been apparent on a contrast enema study.

**Table 1:** Timeline of clinical presentation and diagnostic progression

Date	Age	Setting	Symptoms	Diagnosis/Treatment
March 29, 2023	5 months 25 days	ED (other hospital)	Cough, respiratory distress	Bronchiolitis → Symptomatic treatment
April 2, 2023	6 months	ED (other hospital)	Fever, ear pain	Acute otitis media → Antibiotics
April 6, 2023 (a.m.)	6 months 2 days	ED (other hospital)	Vomiting, oral intolerance	Suspected ATB intolerance → IV fluids, antiemetic drugs
April 6, 2023 (p.m.)	6 months 2 days	ED (transferred)	Persistent vomiting, blood-streaked stools	Acute gastroenteritis → IV antibiotics for AOM
April 8, 2023	6 months 4 days	ED	Recurrent vomiting after meals	Gastroenteritis
April 9, 2023	6 months 5 days	ED	Persistent vomiting, poor oral intake	IV fluids, antiemetic drugs → Readmitted after vomiting upon discharge
April 10–17, 2023	6 months 6–13 days	Hospitalization	Postprandial vomiting, progressive improvement	Adenovirus isolated → Supportive care
April 29, 2023	6 months 25 days	ED	Persistent vomiting, weight loss, occipital pain (normal head CT)	Outpatient referral
May 1, 2023	7 months	ED	Recurrent vomiting after all meals	Ileocolic intussusception on abdominal US → Surgical referral

Abbreviations: AOM, acute otitis media; CT, computed tomography; ED, emergency department; IV, intravenous; US, ultrasound.

Intraoperative findings included the appendix in the left iliac fossa in laparoscopy, and after conversion to an infraumbilical mini-laparotomy, a resolved ileocecal intussusception, likely recent, with thickened, umbilicated cecum, multiple mesenteric lymphadenopathies near the terminal ileum, and Ladd's bands causing duodenal compression. The Ladd procedure was performed, including band lysis, mesenteric widening, appendectomy, and repositioning of bowel loops in a non-rotated orientation.

The postoperative course was uneventful. The patient gradually resumed oral intake and was discharged in good condition on the third postoperative day. Follow-up visits over 2 years showed normal weight progression and no recurrence of gastrointestinal symptoms.

## Discussion

This case illustrates the diagnostic challenge of intestinal malrotation when presenting with non-specific symptoms such as recurrent vomiting, feeding intolerance, and weight loss. The persistence of symptoms led to multiple emergency visits before the development of intussusception prompted further investigation. Notably, malrotation was suspected preoperatively based on ultrasound findings, an achievement rarely documented in the literature. This highlights the importance of maintaining a high index of suspicion for malrotation in atypical or recurrent cases of intussusception.

Waugh's syndrome, the coexistence of intussusception and malrotation, is rare but likely underrecognized. Although both conditions are common in pediatrics, their concurrence is reported infrequently, suggesting missed diagnoses in cases managed non-operatively. Surgical management in our case, involving reduction of intussusception followed by the Ladd procedure, reflects current practice and aligns with previous reports. 1 7

The association was first described by Waugh and Lond in 1911, 2 and Brereton et al later conducted the only prospective study to date, finding malrotation in 40% of intussusception patients. 8 Subsequent reports have reinforced that the condition may be more common than recognized, including 7 of 106 cases 9 and 10 of 138 cases younger than 2 years. 10 However, malrotation is usually identified intraoperatively, as non-operative reduction often suffices, and no further evaluation is pursued. 4 10 The pathophysiology is thought to involve non-fixation and increased mobility of the cecum and ileum, predisposing to telescoping. 1 Although Waugh's syndrome is predominantly a pediatric entity, with most cases reported in infants and young children, a small number of adult cases have also been documented. These adult reports highlight that the condition may persist into later life if unrecognized, but the clinical presentation and diagnostic challenges remain distinct from those in the pediatric population. For clarity, in our review, we categorized adult cases separately while focusing on the pediatric presentation as the core of this syndrome.

**Table 2:** Literature summary of Waugh's syndrome cases

Study	Year	Journal	New cases	Cumulative	Note
Waugh and Lond 2	1961	Lancet	1	1	First reported case
Breckon and Hadley 1	1999	British Journal of Surgery	6	39	Included 33 prior + 6 new; named the syndrome
Baltazar et al 4	2012	International Journal of Surgery Case Reports	1	56	Some miscounting; total claimed as 54
Zavras et al 3	2016	Case Reports in Surgery	1	82	Included all reported by Zavras et al 3 and Baltazar et al 4
Newly listed					
Hsieh et al	2008	Digestive Diseases and Sciences	1	–	Adult case
Chaudhary et al	2012	Clinics and Practice	1	–	Adult case
Behera et al 12	2014	Journal of Clinical and Diagnostic Research	1	–	–
Saxena et al	2015	Pediatrics and Neonatology	1	–	Neonatal case
Khan et al 11	2017	European Journal of Pediatric Surgery Reports	2	–	–
Kumar et al	2017	Clinical Pediatrics Open Access	1	–	–
Siviero et al	2017	Journal of Case Reports	1	–	–
Tovar et al	2018	Revista Cubana de Cirugía	1	–	Spanish article
Ahmad et al	2019	Journal of Surgery and Trauma Care	1	–	–
Ghandi et al	2019	BMJ Case Reports	1	–	Adult case
Induchoodan et al	2020	Indian Journal of Surgery	1	–	Adult case
Sousa et al	2020	Revista Española de Enfermedades Digestivas	1	–	First Portuguese report; adult case
Dirani et al	2021	Clinical Images and Case Reports Journal	1	–	–
Emeka et al	2021	Journal of Medical Case Reports and Case Series	1	–	Presented with transanal protrusion of intussusception
Saxena et al	2021	Pediatric Emergency Care	1	–	
Manekar et al	2021	Annals of Pediatric Surgery	1	–	–
Carmine et al	2022	Journal of Pediatric Surgery Case Reports	1	–	Presented with TAPI
Elkeir et al 7	2022	International Journal of Surgery Case Reports	4	–	Pediatric case series
Govani et al	2023	SMP Child Health and Pediatrics	1	–	–
Ahmed et al	2024	Journal of Medical Case Reports	2	–	–
Asbah et al	2024	International Journal of Surgery Case Reports	1	–	Preoperative diagnosis with CT
Afasha et al	2024	Cureus	1	–	Adult case with cystic fibrosis
Thakur et al 10	2024	Journal of Indira Gandhi Institute of Medical Science	10	–	Review article of 133 intussusception patients, aged more than 2 years
Correia et al	2025	European Journal of Pediatric Surgery Reports	1	**120**	–
Excluded					
Sinha et al	2013	BMJ Case Reports	1	–	Lymphoma as a pathological lead point
Singh et al	2014	Journal of Case Reports	1	–	Meckel's diverticulum as PLP
Tripathy et al	2016	Journal of Clinical and Diagnostic Research	1	–	PLP
Kubo et al	2019	Pediatrics International	1	–	Postoperative intussusception
Tamasia et al	2020	Journal of Radiological Review	1	–	No access to abstract/article; Potential PLP suggested by title
Trambadia et al	2022	Indian Journal of Pediatrics	1	–	No access to abstract/article

Abbreviations: PLP, pathological lead point; TAPI, transanal protrusion of intussusception.

While a high index of suspicion for malrotation is essential in infants with recurrent vomiting or failure to thrive, preoperative recognition of malrotation in the specific setting of Waugh's syndrome remains exceptional. In Zavras et al's 2016 review, only seven cases had a definite preoperative diagnosis based on imaging. 3 Although preoperative suspicion of malrotation on ultrasound has become increasingly reported in recent pediatric radiology literature, such recognition remains uncommon in cases of Waugh's syndrome, where the overlapping presentation of intussusception often obscures the underlying anomaly. Our case, therefore, adds to this small but growing subset, emphasizing the critical role of expert radiology in recognizing abnormal colonic configuration and mesenteric vessel orientation.

The true incidence of Waugh's syndrome remains uncertain, but literature reviews suggest approximately 120 cases worldwide to date ( Table 2 ). 1 3 4 Despite the accumulation of reports, no prospective studies have been conducted since Brereton et al's work in 1986, and no guidelines exist to direct clinicians on when to suspect malrotation in intussusception. This represents a major gap in pediatric surgical knowledge.

Given these limitations, careful diagnostic workup should be considered in recurrent or atypical intussusception. Ultrasound, already the standard for diagnosing intussusception, can also provide valuable information on bowel position and vessel orientation, potentially identifying malrotation before surgery. Early recognition of malrotation may guide timely operative management, as hydrostatic reduction alone is insufficient when both pathologies coexist.

Reduction of the intussusception followed by the Ladd procedure remains the gold standard. 11 12 While open surgery is the most common approach, laparoscopy may offer a safe and effective alternative; however, only one laparoscopic case has been reported to date. 13 In our case, the procedure was initiated laparoscopically, which allowed confirmation of both the intussusception and the previously suspected malrotation. This approach underscores the value of laparoscopy as a diagnostic adjunct in complex or atypical presentations, even when definitive management may ultimately require conversion to laparotomy.

In summary, this case highlights the rare but significant coexistence of intussusception and malrotation. Its key contribution lies in the preoperative suspicion of malrotation based on ultrasound, an achievement documented in fewer than 10 cases worldwide. Heightened awareness, combined with improved radiologic expertise, can allow earlier diagnosis and optimal surgical intervention. Further prospective studies are needed to establish incidence, diagnostic criteria, and management guidelines for this underrecognized syndrome.

## Notes

**Importance for the Pediatric Surgeon**
Waugh's syndrome, though reported fewer than 100 times, may be significantly underdiagnosed due to the frequent non-operative management of intussusception. This case highlights the critical need for pediatric surgeons to maintain a high index of suspicion for malrotation in atypical or recurrent intussusception. Preoperative imaging has also evolved and may be suggestive of abnormal bowel positioning that should prompt more investigation. Early recognition and definitive treatment, including the Ladd procedure, can prevent complications such as recurrence, volvulus, or bowel ischemia. Awareness of this association can directly impact surgical decision-making and patient outcomes.
